# Expenditure on Infant Formula, Brand Decisions and Misleading Marketing in the UK: Inequitable Impacts Upon Low‐Income Families

**DOI:** 10.1111/mcn.70296

**Published:** 2026-11-11

**Authors:** Amy Brown, Charlotte Walker, Aimee Grant, Sally Etheridge, Sabine Goodwin, Catrin Griffiths, Sara Jones, Holly Morse, Rhiannon Phillips, Rosalind Sharpe, Victoria Thomas, Victoria Sibson

**Affiliations:** ^1^ Faculty of Medicine, Health and Life Sciences Swansea University Swansea UK; ^2^ Centre for Lactation, Infant Feeding and Translation (LIFT) Swansea UK; ^3^ Leicester Mammas Leicester UK; ^4^ Independent Food Aid Network London UK; ^5^ Cardiff Metropolitan University Cardiff UK; ^6^ University of Hertfordshire Hatfield UK; ^7^ Newcastle Upon Tyne Hospitals NHS Foundation Trust Newcastle UK; ^8^ First Steps Nutrition Trust, The Food Exchange London UK

**Keywords:** advertising, conflicts of interest, financial difficulties, food insecurity, greenwashing, infant feeding, infant formula, marketing, poverty

## Abstract

Infant formula brands in the United Kingdom (UK) differ significantly in cost despite no evidence of preferential impacts upon infant health and development. This mixed methods study explored the factors that drive higher spending on formula milk, comparing this for families who were struggling to afford formula milk or not. UK parents with a baby aged 0–12 months who use infant formula completed an online survey (*n* = 478). Additionally, 20 parents who were struggling to afford infant formula taking part in interviews. Expenditure and brand decisions were compared for those struggling to afford infant formula and eligible for financial support (*n* = 94), struggling but not eligible for support (*n* = 167) and not struggling to afford infant formula (*n* = 217). There was no significant difference in weekly amount spent between the three groups. Two thirds of parents chose higher‐cost brands although mothers who were mixed feeding were less likely to buy a higher‐cost milk than those exclusively formula feeding. Parents who were struggling to afford formula were more likely to buy a brand due to perceived better‐quality/additional ingredients and because it was supplied in hospital and perceived to have healthcare professional endorsement than those who were not struggling. Qualitative analysis showed that parents bought higher‐cost brands in case future research showed an impact, from fear of judgement, worries around brand availability, and perceptions that brands were closest to breast milk. Stronger regulation is needed of misleading marketing of breastmilk substitutes alongside ensuring availability and accessibility of lower‐cost products.

## Key Messages

- Parents who struggle to afford infant formula are more likely to purchase higher priced milks than those who are not struggling due to perceptions of superior ingredients or endorsement by healthcare professionals due to provision in healthcare settings.
- Anxiety that they might be judged for using lower cost products drives some parents to buy higher priced milks.
- Some parents are suspicious of lower costs milks and feel that higher priced milks might use better quality ingredients.
- Some mothers who have been unable to breastfeed may be vulnerable to misleading messaging around certain brands being closer to breast milk.

## 1 Introduction

For parents who use infant formula, deciding which brand to use and how much to spend can feel like a complex issue. All infant formulas (suitable from birth to 12 months) for sale in the United Kingdom (UK) have comparable nutrition due to strict production regulations (EUR‐Lex 2016). There are no established differences in infant health or developmental impacts between infant formula brands (EFSA Panel on Dietetic Products, Nutrition and Allergies NDA 2014). Despite this, the cost of products varies considerably (First Steps Nutrition Trust 2025) with products available at the time of writing ranging from £6.99–£18.

There are many drivers of brand choice, with beliefs about certain brand superiority common. In the UK, regulations prevent infant formula (suitable from birth to 12 months) from being marketed to parents. However, marketing is allowed for other formula milk products labelled as suitable from 6 to 12 months (“follow on” milks) or 12 months plus (“toddler”/“growing up” milks). Brand recognition means that parents can infer that messaging surrounding follow on or toddler milks also applies to infant formula or assume that it is infant formula being advertised (Rollins et al. 2023; Conway et al. 2023). Misleading marketing strategies imply that certain brands have a greater positive impact on infant health, behaviour or development compared to other brands (Rollins et al. 2023; Hastings et al. 2020). Highly marketed products are often more expensive, in part due to advertising spend, leading parents to pay more for products out of the belief that they will promote infant health or development (Munblit et al. 2020; van Tulleken et al. 2020; Romo‐Palafox et al. 2020).

Parents also sometimes perceive that brands are being endorsed or recommended by health professionals. When specific brands are offered in postnatal settings, this can be perceived as the “best” milk, with parents continuing to use it post‐discharge (Brown et al. 2020; Vilar‐Compte et al. 2024). This can be exacerbated by some parents believing it is risky to change brands/products once a baby is settled on one (Conway et al. 2023), although there is no evidence for this.

Purchasing a brand that is perceived as “best” can sometimes help ease parental anxiety or guilt (Conway et al. 2023). Infant feeding experiences can be highly emotionally charged, due to feelings around judgement, guilt and a lack of support leading to feeding decisions that mothers are often distressed by (Brown 2018; Doughty et al. 2025). Desire to purchase a formula brand that is “closest to breast milk” or “best” can be strong amongst mothers who wanted to breastfeed but had to stop before they were ready (Brown et al. 2026).

This is problematic because some parents may be spending more than is necessary or they can afford on formula milk, not through consumer choice but from a belief that higher cost products will have a preferential impact for their baby. In recent years infant formula prices have increased over and above already high food inflation costs (Borland 2026), rising by an average of 24%, with increases of up to 45% between March 2021 and April 2023 (First Steps Nutrition Trust 2023). Infant formula milk is an expensive product even before marketing encourages purchasing of higher priced products. Profits are significant. The recent Competition and Markets Authority's (CMA) infant formula and follow‐on formula market report estimated manufacturers' variable gross margin was calculated at between 50% and 75% in 2024 and retail gross profit margins between 18% and 22% (Competition and Markets Authority 2025).

Numerous recent articles in the UK press have highlighted how the rising costs of infant formula milk have caused financial difficulties and distress for some parents (BBC News 2023; The Guardian 2023). Our analysis of the impact of infant formula pricing on families indicated that UK parents who struggled to afford infant formula reported considerable distress, guilt and anxiety, with some resorting to unsafe feeding practices such as watering down feeds or using leftover milk for later feeds. Many sourced products from social media marketplaces, used milks marketed for babies/young children older than their own baby, or were accumulating debt in order to buy higher cost brands (Brown et al. 2026).

Parents experiencing financial difficulties might be differentially impacted by marketing and perceived endorsements. They are more likely to use formula milk (Quigley 2006), partly influenced by cultures of lower levels of breastfeeding in lower income communities and increased difficulty in finding support (Hardison‐Moody et al. 2018). Coined the “Breastfeeding paradox” women on a low income, who could benefit the most financially from being able to breastfeed, often face the most social, cultural and economic barriers to doing so (Frank 2015). Mothers on a lower income may also be more likely to feel powerless and worried about the impacts of poverty upon their baby (Gross et al. 2022) and worry about doing the “best” for their baby to make up for the impacts of poverty (Rose and McAuley 2019). This can be especially true if they feel under scrutiny from health and social care professionals and judged more harshly than parents from higher socioeconomic backgrounds (Mannay et al. 2018).

The aim of this analysis was to explore how much parents are spending on formula milk and the factors that drive brand decisions, as well as potential differences according to socio‐economic status that could influence inequalities in product choices/impact of these choices.

## 2 Methods

### 2.1 Design

A mixed‐methods study using a cross‐sectional online survey and semi‐structured interviews with UK parents who use formula milk. This data are part of a larger study exploring the impact of high infant formula milk prices upon feeding practices and parental wellbeing. The larger study included the views of 642 UK parents with a baby aged 0–12 months old and 158 UK professionals and volunteers working in infant feeding, parenting and food poverty support. Here we report unique findings from a subset of this sample (*n* = 478) who used formula milk, specifically in relation to formula milk expenditure, brand decisions and brand influences. The remaining 164 parents were not included in this analysis because they did not have experience of using formula milk. The data reported in this analysis are not included in any other paper related to the study.

Please note, in the introduction we refer to “infant formula milk” to describe milks suitable from 0 to 12 months. This is the only type of formula milk recommended by the NHS (National Health Service 2023). However, parents often reported using milks marketed at older babies (e.g. “follow on” or “toddler” milks, neither of which are recommended products (National Health Service 2023). To manage this, we use the term ‘formula milk’ throughout the paper unless explicitly referring to infant formula.

### 2.2 Participants

Parents who had experience of giving their baby infant formula. Inclusion criteria were UK‐based, parent aged 16+ , baby age 0–12 months, using formula milk (any type) partially alongside breastfeeding or exclusively, able to complete the survey in the English language and able to give informed consent.

For the survey parents were eligible to take part if they met the inclusion criteria regardless of income. For the interviews, parents had to meet inclusion criteria plus be experiencing significant difficulties in affording infant formula for their baby. We used the following definition in the survey to split parents into financial groups (struggling or not struggling) and the same definition for recruitment to interviews.

Have you ever found yourself struggling to afford to buy formula milk but managed to do so by making significant cuts elsewhere such as going without food yourself, not being able to heat your home, not paying a bill, taking on extra work or getting into debt? Or you've had to ask for help (from family, friends, a health professional, food bank or charity) so your baby doesn't go without?

This definition was used rather than income level because many media articles have highlighted families may struggle to afford formula milk due to the increased cost of living (i.e. housing, utilities, food costs, childcare) despite not having the lowest incomes to qualify for financial support.

Full ethical permission for the study was gained from Swansea University School of Health and Social Care Research Ethics Committee. Participants gave informed consent, and all aspects of the study were carried out in line with the Declaration of Helsinki (2024).

### 2.3 Measures

Parents completed an online survey containing both closed and open questions, hosted by Qualtrics. Data relevant to this study included parent demographic background, feeding methods and decisions on which brand of formula milk to buy. This included closed item questions on:

- How babies were fed including which milks were given (breast, formula, cow's milk) and introduction of solid foods [tick box for all that apply].
- Brand of formula used [open text box].
- Type of formula used e.g. powdered or ready to feed.
- Typical cost of formula milk per week [estimated in pounds].
- Whether parents struggled to afford other aspects of preparing formula e.g. teats, sterilising, bottles [Strongly agree – strongly disagree 5 point likert scale].
- Reasons for choosing formula milk brands [Yes/no] (see Table 4 for a full list).

Reasons for choosing formula milk brands were based on influences identified by previous research [4–12] and developed with our study team (including those with backgrounds in midwifery, health visiting, paediatrics, and infant nutrition alongside our parent PPI members). Parents could select as many as they applied. We also included open‐ended boxes to expand on reasons given or additional reasons, alongside space for reflection on experiences of choosing and using formula milks, and “any other comments.”

Additionally, we asked parents whether they claimed Healthy or Best Start allowance. In the UK, Healthy Start (England, Wales and Northern Ireland) and Best Start (Scotland) allowance provides pregnant women or parents of a child under 4 who are on a low income with a pre‐paid card for purchases of healthy foods such as cow's milk, fruits, vegetables and pulses and infant formula. The earnings cut‐off at the time of the survey to qualify was £408 per month. This therefore approximates low income, since not all parents who are eligible for Healthy/Best Start are aware of it or know how to claim it (and would therefore not be categorised in the Healthy/Best Start group). Additionally, some parents may be on a low income but not be eligible for recourse to public funds. It is therefore an approximate measure. The survey contained details of how they could find out more information if they felt they were eligible.

Interviews were conducted with parents who were struggling to afford formula milk. These interviews were part of the larger study and explored parents' experiences of buying or accessing infant formula for their baby, how they coped, how high formula prices made them feel and what they thought the solutions to ensuring parents experiencing financial difficulties could access formula might be. Relevant interview questions for this study included items that specifically asked about which brands of milk they bought and the reasons behind their choice. However, sometimes in the wider interview questions parents talked about brand perceptions, preferred products and influences on brand choices. Therefore, sometimes data was used from these wider questions.

### 2.4 Procedure

Data were collected between March and August 2025. Separate study advertisements for the survey and interviews were shared on social media with encouragement for others to share. Advertisements contained information about the study and inclusion criteria. Posts were shared on the academic/organisational pages of the diverse study team (academic, infant feeding, child health, parenting and food bank roles), including Facebook, Instagram, LinkedIn and X. The study team has a wide social media reach and with sharing of posts sufficient participant numbers (based on population sample size calculations) and diversity of interview participants (in terms of age of baby, feeding approach and ethnicity) was reached without needing paid posts. Hashtags such as #feedingbabies #costofliving #parenting #money were used to distribute the survey wider than connected accounts. Team member organisations working in infant feeding, parenting and food bank support also shared the post on their social media pages, with their mailing lists and face‐to‐face, e.g. in support groups.

The survey study advertisement contained a link to the study information sheet. Consent questions were followed by the survey if the participant consented. Once completed, a debrief statement was given, including contact details for support organisations. Participants could also leave an expression of interest to take part in an interview. Paper copies of the survey were offered during face‐to‐face groups (or if an online participant wished to be sent one, but no one requested this option).

For the interview advertisement, an expression of interest procedure was used. Potential participants read through the study information sheet and then if they wished to proceed, answered demographic questions and provided contact details. A purposive sample of participants was invited to interview (aiming for a diversity of age, education, and ethnicity), including sending the study information sheet again, consent questions and an option to confirm they would like to take part. An interview was then arranged. Interviews were conducted and recorded via MS Teams or Zoom, with one interview conducted over the telephone due to technical difficulties. To support parents who might not be able to afford digital access, a pre‐paid SIM card was an option. Face‐to‐face interviews were advertised but all participants preferred an online format, often due to childcare and accessibility.

At the start of the interview participants were reminded of the study aims and procedures and asked to reconfirm consent, noting that they could stop at any time and did not have to answer any questions they did not want to. Participants could turn their camera off during the recording, after initial introductions (which were not recorded). Participants could discuss topics in whichever way was most comfortable, aiming to cover the areas in the topic guide. At the end of the interviews, participants were debriefed and given information about support organisations. They received a £20 shopping voucher for their time.

### 2.5 Reflexivity Statement

Our team consisted of academics, health professionals and charity organisations working in infant feeding and food bank support. Members came from backgrounds including midwifery, health visiting, social sciences, psychology and public health alongside external organisation members in infant nutrition, food insecurity, food systems, infant feeding support and paediatrics. Between us we have significant experience in conducting research, and supporting parents directly, with infant feeding and wellbeing, often with marginalised or underserved groups. Several of the team members had experience of feeding their own babies, with a diverse combination of feeding experiences between the team. Some team members had their own experience of financial difficulties, either more broadly during their lives or specifically as a new parent, including experiencing negative attitudes in relation to socioeconomic status.

### 2.6 Data Analysis

For the survey data, quantitative data were analysed using SPSS version 29. Descriptive analysis was carried out on the closed‐response survey data to provide frequencies of response categories, e.g. for brands listed and reasons for brand usage. Mean expenditure was calculated for the whole sample, by partial/exclusive formula feeding, and baby age.

In terms of brands used, nine brands were listed by parents. Brands were split into higher or lower cost brands using a price point of </> £10.50 for a standard 800 g tub. We used a report published by the charity First Steps Nutrition Trust which had calculated prices of infant formula milks in 2025 to categorise brands into these two groups (First Steps Nutrition Trust 2025). This price point split placed five brands in the lower cost group and four in the higher cost group. It allowed two supermarket own brands plus two lower cost widely available newer “budget” options made by established brands to be placed in this category. Additionally, we included another established brand in this category that has historically been marketed as a milk for parents who want to see their baby happy and is lower than brands that tend to be marketed in relation to ingredients (Hastings et al. 2020). It is also the lowest cost brand that is typically available in most local shops (not budget supermarkets) and may therefore be the lowest cost brand parents can access if they do not have transport. We also created an additional group called “lowest cost” brands (two supermarket own brands, the lowest cost available on the market at the time of data collection) to examine how many parents purchased these brands.

Based on these, the two questions that asked whether parents were struggling to afford infant formula (yes/no) and whether they were accessing Healthy/Best Start (yes/no), we categorised parents into three financial groups: Not struggling to afford formula, struggling but not eligible for Healthy/Best Start, struggling and claiming Healthy/Best Start. This allowed us to demonstrate how parents who fall outside the qualifying income criteria for Healthy/Best Start can still be experiencing significant financial difficulty due to high cost‐of‐living expenditure on basics and essentials. No parent reported that they were not struggling but accessing Healthy/Best Start. Differences in expenditure, product choices and reasons for choosing products were compared using MANCOVA (controlling for parent age, education, disability and neurodivergence) or chi‐square for the three financial groups.

To explore in depth the experiences of parents who were struggling financially, we combined data from the interviews and open‐ended boxes in the survey described in the measures. Interviews were transcribed, and a simple descriptive approach (Sandelowski 2010) was used to conduct a thematic analysis (Proudfoot 2023). A deductive coding approach was followed to explore decisions around brand and price, specifically exploring the data to identify reasons for brand use based on the factors identified for the survey (see Table 4 for a full list). Further inductive codes were generated from the data for additional themes that had not been anticipated prior to analysis (e.g. the impact of breastfeeding difficulties, ethical influences).

To minimise bias, principles of trustworthiness were followed, considering the criteria of credibility, transferability, dependability and confirmability (Lincoln and Guba 1985). One author (CW) immersed themselves in the data, reading through responses from each participant and across questions for all participants and re‐reading them iteratively to develop themes. To enhance reliability of the analysis, another author (AB) reviewed proposed themes and subthemes. Where disagreement occurred, themes would be discussed until agreed, although no significant disagreement occurred. The proposed themes were also shared and discussed with the wider research team.

### 2.7 Ethics Statement

This study was conducted according to the guidelines laid down in the Declaration of Helsinki, and all procedures involving research study participants were approved by the School of Health and Social Care Research Ethics Committee, Swansea University. Written informed consent was obtained from all subjects/patients.

## 3 Results

Overall, 478 parents completed the survey (Table 1), and 20 parents who were experiencing financial difficulties took part in interviews (Table 2). The majority of participants were mothers, but our sample included fathers and therefore we refer to “parents” to refer to broad participant groups, with mother or father for specific quotes.

**Table 1:** Parent demographic background for survey participants split by financial group.

		Full sample (*n* = 478)		Accessing healthy start (*n* = 94)		No healthy start (*n* = 167)		Not struggling (*n* = 217)	
**Category**	**Sub‐category**	**N**	**%**	**N**	**%**	**N**	**%**	**N**	**%**
Parent	Mother	468	97.9	93	98.9	165	98.8	210	96.8
	Father	10	2.1	1	1.1	2	1.2	7	3.2
Gender	Same as sex	470	98.2	91	96.8	165	98.8	214	98.6
	Fluid	1	0.2	1	1.1	0	0.0	0	0.0
	Non binary	4	0.8	1	1.1	1	0.6	2	0.9
	Transgender	2	0.4	1	1.1	1	0.6	0	0.0
	No gender identity	1	0.2	0	0.0	0	0.0	1	0.5
Age	18–24	90	18.6	31	31.9	44	26.3	15	6.9
	25–29	97	20.5	29	31.9	41	24.6	27	12.4
	30–34	159	33.3	20	21.3	52	31.1	87	40.1
	35–39	99	20.7	10	10.6	26	15.6	63	29.0
	40+	33	6.9	4	4.3	4	2.4	25	11.5
Education	No formal qualifications	9	1.9	5	5.3	4	2.4	0	0.0
	GCSE or equivalent	60	12.6	23	24.5	30	18.0	7	3.2
	A level or equivalent	170	35.6	41	43.6	88	52.7	41	18.9
	Degree or equivalent	125	26.2	17	18.1	37	22.2	71	32.7
	Postgraduate qualification or equivalent	113	23.6	8	8.5	8	4.8	97	44.7
Ethnicity	Asian or Asian British: Bangladeshi	13	2.7	1	1.1	8	4.8	4	1.8
	Asian or Asian British: Chinese	10	2.1	2	2.1	2	1.2	6	2.8
	Asian or Asian British: Indian	28	5.9	6	6.4	14	8.4	8	3.7
	Asian or Asian British: Pakistani	24	5.0	11	11.7	8	4.8	5	2.3
	Any other Asian background	2	0.4	0	0.0	1	0.6	1	0.5
	Black or Black British	38	7.9	7	7.5	14	8.4	17	7.9
	Mixed or Multiple	56	14.5	8	8.5	14	8.4	46	9.6
	White British or Irish	366	57.7	53	56.4	95	56.9	128	59.0
	White (other)	29	6.1	6	6.4	10	6.0	13	6.0
	Gypsy or Irish Traveller	1	0.2	0	0.0	1	0.6	0	0.0
	Any other group	9	1.9	0	0.0	0	0.0	9	4.1
	Prefer not to say	2	0.4	0	0.0	0	0.0	2	0.9
Number of children	One child	210	43.9	48	51.1	78	46.7	84	38.7
	More than one child	268	56.1	46	48.9	89	53.3	133	61.3
Country	England	310	64.9	55	58.5	103	61.7	152	70.0
	Northern Ireland	2	6.1	10	10.6	10	6.0	9	4.1
	Scotland	42	8.8	7	7.4	11	6.6	24	11.1
	Wales	97	20.3	22	23.4	43	25.7	32	14.7
Disability or chronic health issue	Yes	80	16.7	31	33.0	27	16.2	22	10.1
	No	398	83.3	203	77.8	140	83.8	195	89.9
Neurodivergence	Yes (including self‐identified)	85	17.8	29	30.8	42	25.2	14	6.4
	Not sure	20	4.2	2	2.1	9	5.4	9	4.1
	No	373	78.0	63	67.0	116	69.5	194	373
Work status	Yes full time	55	11.5	0	0.0	39	23.4	16	7.4
	Yes part‐time	96	20.1	27	28.7	42	25.1	27	12.4
	Yes zero hours	10	2.7	4	4.3	0	0.0	6	2.8
	Maternity/paternity leave	272	56.5	29	28.7	81	48.5	162	74.7
	No	45	9.2	34	38.3	5	1.2	6	2.8
Live with a partner	Yes	395	82.6	40	42.6	155	86.8	200	97.7
	No	83	17.4	54	57.4	12	13.2	17	2.3
Partner in work (*n* = 395)	Yes full time	281	75.1	0	0.0	108	78.2	173	87.3
	Yes part time	44	5.8	2	5.0	30	6.3	12	5.7
	Yes zero hours	13	4.1	7	17.5	4	4.9	2	0.9
	Maternity/paternity leave	11	3.3	4	7.5	0	2.1	7	3.3
	No	46	11.4	28	70.0	12	8.4	6	2.8

**Table 2:** Parent demographic background for interview participants (using pseudonyms).

Pseudonym	Age	Ethnicity	Baby age	Feeding method
Hedi	25–34	White	12 weeks	Formula milk
Zoe	25–34	White	11 days and 12 m	Formula milk
Rosie	25−34	White	8 months	Formula milk
Olivia	25−34	White	7 months	Mixed feeding
Leila	25−34	Asian	6 months	Formula milk
Abeni	—	Black/African/Caribbean	8 months	Formula milk
Holly	25−34	White	4.5 months	Mixed feeding
Emma	25−34	Black	8 months	Formula milk
Nicola	35−44	White	11weeks	Formula milk
Lara	35−44	White	5 months	Formula milk
Marie	25−34	Black/African/Caribbean	12 months	Mixed feeding
Lisa	35−44	White	19 weeks	Formula milk
Malik	35−44	White	7 months	Formula milk
Katie	35−44	White	4 months	Formula milk
Lesley	25−34	White	4 months	Formula milk
Catrina	25−34	Black/African/Caribbean	5 months	Mixed feeding
Alayah	—	Asian	9 months	Formula milk
Lucy	25−34	Black/African/Caribbean	7 months	Formula milk
Aditi	—	Asian	7 months	Mixed feeding
Alice	25−34	White	4 months	Formula milk

Our sample included 261 (54.6%) parents who reported struggling to afford infant formula and 217 (45.4%) who did not. Splitting the ‘struggling’ group further, this meant that 94 (19.7%) of the sample were claiming Healthy/Best Start, 167 (34.9%) were struggling but not eligible/unaware of Healthy/Best Start, and 217 (45.4%) were not struggling.

Significant differences were found between these groups for parent age [F (2, 475) = 39.191, *p* < 0.001]. Post hoc Bonferroni tests found that those who were not struggling were significantly older than both the struggling groups (*p* = < 0.001). A significant association was also found for education group [*χ* ^2^ = 155.286, *p* < 0.001] with 26.6% of those in the Healthy/Best Start group, 27.2% in the struggling but not accessing Healthy/Best Start and 49.8% in the not struggling group having a degree‐level or above qualification.

Disability or chronic health condition was also significant [*χ* ^2^ = 24.163, *p* < 0.001], with 38.8% in the Healthy/Best Start group, 33.8% in the struggling but no Healthy/Best Start group and 27.5% in the not struggling group having a disability or chronic health issue. Finally, a significant association was found for neurodivergence [*χ* ^2^ = 42.308, *p* < 0.001], with 30.8% in the Healthy/Best Start group, 25.2% in the struggling but no Healthy/Best Start group and 6.4% in the not struggling group considering themselves to be neurodiverse (diagnosed or self‐identified).

For baby age, 60.8% (*n* = 290) had a baby aged 0–6 months and 39.2% (*n* = 187) had a baby over 6 months. This is important to measure because after around 6 months of age babies would be expected to reduce formula intake (and therefore expenditure) gradually as solid foods are introduced. There was a significant association between baby age and financial group (*χ* ^2^ = 11.466, *p* = 0.003). Babies aged 0–6 months made up 66.7% (*n* = 62) of the Healthy/Best Start group, 68.3% (*n* = 114) of the struggling but not accessing Healthy/Best Start group, and 52.5% (*n* = 114) of the not struggling group. Parents who were not struggling were less likely to have a baby aged 0–6 months.

### 3.1 How Were Babies Fed?

All babies were formula fed; 209 (43.7%) also had breast milk, 267 (55.9%) solid foods, and 42 (8.8%) cow's milk as a drink. We refer to those who only had formula milk as exclusively formula fed and those who were receiving formula milk alongside breastmilk as being partially formula fed. Overall, 209 babies were partially formula fed (43.7%) and 269 (56.4%) were exclusively formula fed.

There was a significant association between partial/exclusive formula use and financial group (*χ* ^2^ = 46.15, *p* = < 0.001). Parents who were struggling to afford formula milk were more likely to have a baby who was exclusively formula fed. Overall, 61.7% (*n* = 58) of those accessing Healthy/Best Start, 74.3% (*n* = 124) of those struggling but not eligible and 40.1% (*n* = 87) of those not struggling were exclusively formula‐fed.

For babies aged younger than 6 months (before the age solid foods are recommended), 90/290 babies (31.0%) had started solid foods. A significant association was seen between starting solids early and financial group (*χ* ^2^ = 8.752, *p* = 0.013). Overall, 37.2% (*n* = 23) of those accessing Healthy/Best Start, 37.7% (*n* = 43) of those struggling but not eligible and 21.1% (*n* = 24) of those not struggling had started solid foods with their baby. Parents who were struggling were more likely to have introduced solid foods before 6 months old.

For babies aged younger than 6 months, 15 (5.2%) had received cow's milk for at least some feeds in place of formula milk. None of the babies in the not struggling group had received cow's milk compared to 6 (9.4%) in the accessing Healthy/Best Start group and 9 (5.4%) in the struggling but not eligible for Healthy/Best Start group.

### 3.2 How Much Do Parents Spend on Formula Milk?

The mean expenditure on formula milk per week for all babies was £11.92 (SD: 4.42) with a range from £4 to £40 (For mean costs see Table 3). This differed significantly between exclusively and partially formula‐fed babies [F (1, 470) = 122.519, *p* < 0.001]. There was no significant difference in cost for babies under 6 months who had started solid foods or not [*t* (287) = 0.970, *p* = 0.876].

**Table 3:** Mean expenditure by feeding, financial and brand groups.

Group	Sub group	Mean spend	SD	Significance
Feeding group	All	£11.92	4.42	[*F* (1, 470) = 122.519, *p* < 0.001].
	Exclusive formula (EF)	£13.62	3.64	
	Partial formula (PF)	£9.71	4.36	
Financial group	Healthy Start	£11.57	4.50	[*F* (2, 469) = 0.555, *p* = 0.575].
	Struggling no Healthy Start	£12.06	3.78	
	Not struggling	£11.97	4.82	
Feeding group ∗ Financial group	EF Healthy Start	£13.72	3.87	[*F* (2, 266) = 0.880, *p* = 0.416].
	EF Struggling no Healthy Start	£13.32	3.31	
	EF Not struggling	£13.99	3.93	
	PF Healthy Start	£8.00	2.96	[*F* (2, 204) = 8.804, *p* = 0.001].
	PF Struggling no Healthy Start	£8.33	2.40	
	PF Not struggling	£10.62	4.90	
Brand type	Higher cost	£13.12	4.50	[*F* (2, 204) = 86.081, *p* = 0.001].
	Lower cost	£9.44	2.96	

When comparing costs by financial group (controlling for parent age, education, disability and neurodivergence), there was no significant difference in amount spent [F (2, 469) = 0.555, *p* = 0.575]. An interaction was found between financial group and exclusive vs partial formula feeding [F (2, 470) = 3.398, *p* = 0.034]. When babies were fully formula fed, there was no significant difference in amount spent between financial groups [F (2, 266) = 0.880, *p* = 0.416]. However, when babies were partially formula fed, a significant difference was found [F (2, 204) = 8.804, *p* = 0.001]. Post hoc Bonferroni tests showed the not struggling group spent significantly more than the accessing Healthy/Best Start (*p* = 0.004) and not accessing Healthy/Best Start (*p* = 0.008) groups.

Parents who were struggling to afford milk (whether accessing Healthy/Best Start or not) were asked how much they felt would be an achievable amount to spend on formula milk per week. The mean perceived affordable cost was £3.50 (SD: 2.74), with a range from £0 to £10. A third of parents (88, 33.0%) stated that £0 was the amount they could realistically afford. However, 21 parents (8%) chose £8 ‐ £9. UK prices at the time of publication would allow two lower cost brands to be purchased within this budget.

### 3.3 Which Brands Do Parents Buy?

Splitting the brands into higher cost and lower cost options (using a price point </> £10.50 for a standard typically 800 g tub), 322 (67.4%) parents used a higher cost brand compared to 156 (32.6%) who used a lower cost brand (Table 4). There was no association between purchasing a higher cost brand or not and whether parents were struggling to afford formula or not [*χ* ^2^ = 0.892, *p* = 0.345].

**Table 4:** Purchasing of higher or lower cost brands milks by financial, feeding and early introduction groups.

		Higher cost brand		Lower cost brand	
		N	%	N	%
Full sample		322	67.4	156	32.6
Financial group	Struggling Healthy Start	57	61.2	36	38.8
	Struggling no Healthy Start	113	67.6	54	32.4
	Not struggling	151	69.5	66	30.5
Feeding group	Exclusive formula	193	71.7	76	28.3
	Partial formula	129	61.7	80	38.3
Early introduction of solids	Yes	62	69.9	28	30.1
	No	129	64.5	71	35.5
Cow's milk as a drink	Yes	7	46.7	8	53.3
	No	184	66.9	91	33.1

Brand decisions varied according to feeding groups. A significant association was found for brand use and feeding group (*χ* ^2^ = 5.376, *p* = 0.020). Those who were exclusively formula feeding were significantly more likely to purchase a higher cost brand (71.7%) than those who were breastfeeding at all (61.7%). For babies aged under 6 months, the association between early introduction of solids, cow's milk as a drink and brand was explored. No significant association was found for either early introduction of solids (yes/no) and brand (*χ* ^2^ = 0.532, *p* = 0.466) or cow's milk as a drink (yes/no) and brand (*χ* ^2^ = 2.592, *p* = 0.107). Splitting the cost of formula milk down further, there were two of the lowest cost brands available at the time of data collection that could be fully covered by Healthy/Best Start allowance. Overall, 46 parents (9.6%) chose these formula milks, 14.9% (*n* = 14) of those accessing Healthy/Best Start, 13 (7.8%) of those struggling but ineligible and 19 (8.8%) of those who were not struggling. There was no significant association between financial group and choosing the lowest cost formula milks (*χ* ^2^ = 0.383, *p* = 0.147). However, when the Healthy/Best Start group were compared to the two other groups combined, there was a significant association between receiving Healthy/Best Start and choosing a lower cost formula milk (*χ* ^2^ = 3.73, *p* = 0.046).

### 3.4 Why Do Parents Purchase Specific Brands?

Parents were asked their reasons for purchasing their chosen brand, with the option to select multiple reasons (Table 5). The most popular reason was specific ingredients of the product (32.6%), although convenience also ranked similarly at 31.6%. Around a quarter of the sample chose “cost.” This was split between those who chose higher cost brands (56.3%) and lower cost brands (43.7%), suggesting that some parents purchase a product because it is higher cost and some because it is lower cost.

**Table 5:** Reasons for choosing a brand of formula milk split by financial and feeding groups.

			Financial group						Feeding group			
	Full sample		Healthy start		Not accessing healthy Start		Not struggling		Exclusive formula use		Partial formula use	
	N	%	N	%	N	%	N	%	N	%	N	%
The ingredients	156	32.6	38	40.4	66	42.0	52	24.0	96	35.7	61	29.2
It's easy to get hold of	151	31.6	28	29.8	59	35.3	64	29.5	86	32.0	65	31.1
The cost^a^	126	26.4	35	37.2	38	22.8	64	29.5	71	26.4	55	26.3
Cost (higher, *n* = 322)	71	22.0	18	31.0	22	19.5	31	20.5	46	23.8	25	19.4
Cost (lower, *n* = 156)	55	35.0	17	47.2	16	29.6	22	33.3	25	32.9	30	37.5
My family tend to use this one	85	17.8	21	22.3	30	18.0	34	15.7	50	18.6	35	16.7
I used it with other babies	78	16.3	19	20.2	27	16.2	32	14.7	42	15.6	36	17.2
It was the one given in hospital	66	13.8	21	22.3	25	15.0	20	9.2	40	14.9	26	12.4
Friends recommended it	60	12.5	15	16.0	18	10.8	27	12.4	33	12.3	27	12.9
A health professional recommended it	54	11.3	13	13.8	15	9.0	26	12.0	33	12.3	21	10.0
No particular reason	44	9.2	3	3.2	17	10.2	24	11.1	15	5.6	29	13.9

^a^ Cost for the full sample and then split by parents who agreed with this reason split by whether these parents used a higher or lower cost milk.

Reasons for using certain brands were compared between the three financial groups (Table 5). Significant associations were found between financial group and using a brand because it was the one given in hospital (*χ* ^2^ = 9.784, *p* = 0.008), for the ingredients (*χ* ^2^ = 12.80, *p* = 0.002), and cost (*χ* ^2^ = 7.263, *p* = 0.026). Table 4 shows that those who were struggling (whether accessing Healthy/Best Start or not) were more likely to select these reasons than those not struggling. In comparison, those not struggling were more likely to select “No specific reason” [*χ* ^2^ = 6.345, *p* = 0.042] than those who were not.

#### 3.4.1 Differences in Brand Decisions Between Exclusive and Partial Formula Use

Brand decisions were compared for those who were exclusively formula feeding or partially using formula milk alongside breastmilk (Table 5). Those who were exclusively formula feeding were significantly more likely to select cost of the milk or the ingredients' [*χ* ^2^ = 4.369, *p* = 0.037] whereas those who were partially formula feeding were more likely to select “no particular reason for choosing this brand” [*χ* ^2^ = 9.693, *p* = 0.002].

### 3.5 Thematic Analysis

Our thematic analysis further explored influences on brand choice for parents in the interviews and survey who were struggling financially. Most parents who described their reasoning or concerns around brand choices tended to explain why they had chosen a higher cost product. Eight reasons for choosing specific brands were identified (with some reflecting closed item options but others moving beyond these). Additionally, a small subset of parents described how they perceived brand equivalence, with no particular reason for using the brand that they did.

#### 3.5.1 Higher Cost Brands Seen as “Better” for Infant Health and Development

Some parents saw higher cost brands as being better for their baby, or having more ingredients, with “*organic”* being a popular reason.

I want my baby to have the brand that is best and I want to give her, even if it is more expensive. I will worry about it later.

(Survey: mother, age 19–24, Bangladeshi, baby 7 months, exclusive formula, not eligible for Healthy/Best Start)

Another perception was that some brands had better quality versions of ingredients in them, even if overall nutritional content was similar.

All formulas have, like the same fat content, the same… but it's what they're putting in to make that fat content, isn't it? And when I've done my research (higher cost brand) always comes up top.

(Interview: Katie, mother, aged 35–44, White, baby 4 months, exclusive formula)

Other parents felt that a lack of evidence of superiority did not prove that there was no impact, worrying that the right study had not been conducted yet, and differences would be found in the future when it was too late.

They say there's no difference between milks but that's only what they know now. What if that changes in future and some were better after all?

(Survey: mother, age 19–24, Chinese, baby 4 months, partial formula, eligible Healthy/Best Start)

The perception of certain brands offering a buffer against the wider impacts of financial difficulties was also described. One mother referred to how she hoped buying a “better” milk would make up for the other things she could not afford to buy her baby.

I may not be able to afford much but buying better milk if I can is one thing I can try to do

(Survey: mother, age 35–39, Bangladeshi, baby 4 months, exclusive formula, not eligible Healthy/Best Start)

#### 3.5.2 Suspicion of Lower Cost Products

Although many participants described how they had been told lower cost milks were equivalent in impact to higher cost milks, some still worried about this. They were anxious that it meant shortcuts or lower quality ingredients had been used.

I've always thought like, obviously Aldi have got their own brand for everything. And I did notice when I was looking on the Internet the other day, that they now do a Mamia, and I think it's like £6.99. But then I wondered like, why is that so much cheaper than the rest of the milks?

(Interview: Lara, mother, aged 25–34, White, baby aged 5 months, exclusive formula)

#### 3.5.3 Guilt Driven Purchasing

Others chose a higher cost milk because they felt guilt or pressure because they were not breastfeeding (or not doing so exclusively).

All these different milks, confusing really as a new mum like which one should you choose? And obviously the guilt of bottle feeding… When I stopped breastfeeding… I researched different milks…I wanted to try and find, like the best formula out there, really like ingredient wise… and (brand) came up the best…Because you want the best for him, don't you? When you stop breastfeeding you feel guilty? That's the problem when you're a mum.

(Interview: Katie, mother, aged 35–44, White, baby 4 months, exclusive formula)

I really wanted to breastfeed and carry a lot of guilt from that which is made worse by struggling to buy her the best

(Survey: mother, age 19–24, Mixed race, baby 3 months, exclusive formula, eligible Healthy/Best Start)

Marketing of milks as “closest to breastmilk’” often drove specific choices

Closest to breastmilk because of the fats

(Survey: mother, age 35–39, White British, baby 8 months, partial formula, eligible Healthy/Best Start)

This also included milks perceived to taste similar to breastmilk

Many mums in a Facebook group recommended it due to tasting the closest to breast milk when I couldn't breastfeed.

(Survey: mother, age 25–29, White British, baby 6 months, partial formula, eligible Healthy/Best Start)

#### 3.5.4 Perceived Endorsement by Healthcare Professionals

Several parents described how specific brands had been “recommended” by health care professionals, often perceiving the brand given in hospital as a recommendation rather than being a selection.

Carried on with the brand recommended in hospital

(Survey: mother, age 19–24, White British, baby 4 months, exclusive formula, eligible Healthy/Best Start)

#### 3.5.5 Wanting Others to Perceive That a “Best” Choice Had Been Made

Some parents wanted others to know that they were using a higher cost formula brand, as they felt they would then perceive them to be making good choices for their baby.

Friends use it and consider it the best and I didn't want to be seen as not prioritising my baby

(Survey: mother, age 35–39, Mixed race, baby 7 months, partial formula, not eligible Healthy/Best Start)

One mother described even moting that they were using a higher cost brand because they were worried others would look down on them for not prioritising it.

I pretend to family that we're using {higher cost brand} as that's what everyone uses but we're not. I keep old tubs out on the counter and even put the {cheaper brand} formula in it. I want people to think I can afford to buy my baby the milk I want.

(Survey: mother, age 19–24, White British, baby 3 months, exclusive formula, eligible Healthy/Best Start)

Fear of reprisals or anxiety about being judged for using a lower‐priced milk also played a role. However, parents who gave this reason did not specify who they were worried would perceive them this way.

I worry I would be judged for buying a cheaper product, that it was a test

(Survey: mother, age 19–24, Black British, baby 3 months, exclusive formula, not eligible Healthy/Best Start)

I would not want to be seen as a mum who buys the cheapest possible just in case

(Survey: mother, age 30–34, Asian Indian, baby 7 months, partial formula, not eligible Healthy/Best Start)

#### 3.5.6 Perceived Health Benefits Are not the Only Driver

Several parents referred to specific milks as being vegetarian and therefore preferable for their vegetarian or vegan family. Another common reason for brand choice was the perceived quality of ingredients in the product, particularly a lack of some ingredients such as palm oil. These examples were only given for higher cost brands. UK production was also favoured.

I like that it is British made, no palm oil or fish oil

(Survey: mother, age 30–34, White British, baby 5 months, exclusive formula, not eligible Healthy/Best Start)

Additionally, some brands were seen to have better ethics than others, with this given as a reason for choosing them even if at a higher cost.

We originally went with {a lower cost} formula because it was affordable. But then, when you look into it, it's still I don't know. I mean, they're all pretty much the same, aren't they? But they had some really good kind of ethics behind their brand. So that's why I kind of went with them in the end.

(Interview: Holly, mother, aged 25–34, White, baby aged 4 months, exclusive formula)

#### 3.5.7 Perception That Some Higher Cost Brands “Suited” a Baby

Some parents described how they felt certain brands suited their baby and helped their baby be more settled. They were then reluctant to switch brands because they worried that their baby would have a bad reaction.

I won't change the brand because my baby likes this one and is settled on it and I like it.

(Survey: mother, age 19–24, Asian Indian, baby 7 months, partial formula, eligible Healthy/Best Start)

Others brought up the idea that it could be harmful to switch brands more than once

My health visitor, she was okay with me changing from (one brand) to (another brand), but having to constantly change, that's not good for her.

(Interview: Lisa, mother, aged 35–44, White, baby 3 months, exclusive formula)

Certain brands were seen as solving specific feeding difficulties with a number of parents giving short descriptions such as “*for cows” milk protein allergy symptoms, ‘reacted to milk*’ or “*was unsettled after feeds*.”

Initially used (cheaper brand) but changed when we were having issues with silent reflux. Although I don't think the milk made a massive difference we just haven't changed back.

(Survey: mother, age 30–34, White British, baby 4 months, exclusive formula, not eligible Healthy/Best Start)

#### 3.5.8 Anxiety That Cheaper Brands Would Run Out or Be Difficult to Find

Security of supply was also a driving factor. One mother described how she was worried that lower cost milks would be harder to get hold of, particularly if the brand was an own‐brand from one specific supermarket. Brands that were higher cost and available in more shops were seen as more reliable.

I think Aldi and Lidl do something like that. It's like their brand of formula. I think one of my friends gets it is really cheap. It's like 8 pounds a tin, but then that would make me anxious that they would run out of it, or there'd be an issue with it. You'd have to go to about 10 different shops to try and get it, or I suppose you'd have to bulk buy.’

(Interview: Lesley, mother, aged 25–34, White, baby aged 4 months, exclusive formula)

### 3.6 Awareness of Brand Equivalence

There was awareness amongst some parents that all infant formula brands were nutritionally equal.

I think people don't realize that like it's all just the same base. For like base ingredients, they get drawn into this like, Oh, this is organic. This has got this in it, not realizing that. Actually, it doesn't need any of that in it. So, they end up paying extortion amounts for no reason.

(Interview: Zoe, mother, aged 245–34, White, babies 11 days and 12 months, exclusive formula)

Some parents had learnt this information from their health professionals and trusted their advice.

I think there's a lot of there's a range of formulas out there and one thing that the health visitors always told me, there's not much different because legally they have to have, you know, the nutrients that are essential for a baby. They all have to have a certain threshold of that.

(Interview: Leila, mother, aged 25–34, Asian, baby 6 months exclusive formula)

Sometimes this information had come too late however which led to anger when parents had previously bought higher cost brands because they had been led to believe that they were superior.

Last baby I used {a higher cost brand} as I was told was the best and I thought the cost mirrored that. When I heard this time that they are all just the same really, I was so angry.

(Survey: mother, age 30–34, White British, baby 5 months, exclusive formula, not eligible Healthy/Best Start)

Others were clear that higher prices and promises around ingredients were simply marketing and a way to increase sales of higher priced products.

It's is a money‐making scheme. I think companies advertise that they for example, I always see {brand} formula ads that you know we've been working with families for over however many years and we care for families and we want to make sure your baby gets the right nutrients. But no, the fact is you're a profitable business and you're trying to make a profit.

(Interview: Leila, mother, aged 25–34, Asian, baby 6 months exclusive formula)

However, some raised an unintended consequence of more parents understanding this and purchasing lower cost brands. This awareness led to an increase in purchasing meaning stocks fell low because of increased demand that was not always consistent.

It's great that there's more awareness that you don't need to spend more money to get a better milk but whenever there's lots on social media about it I then find it hard to buy the milk we rely on. I think better off parents decide that it's ok for their baby and shops can't keep up. They can buy whatever milk they want obviously but it ends up being unfair on people like me who don't just have a choice to spend more.

(Survey: mother, age 19–24, White British, baby 3 months, exclusive formula, not eligible for Healthy/Best Start)

## 4 Discussion

Our study explored how much parents in the UK who use formula milk to feed their babies are currently spending per week and the reasons behind purchasing different priced products. In our quantitative data, two‐thirds of parents purchased higher cost brands, driven by factors such as additional ingredients, higher cost milks being perceived as better quality and continuing to use brands supplied in hospital settings. In our qualitative findings, parents experiencing financial difficulties described how they were anxious about being seen to buy what they viewed as ‘better’ brands, were suspicious of lower cost products, or worried that differences in impact would be discovered in the future. Although our findings are observational and cross‐sectional, they have important implications for those supporting parents and for regulators determining appropriate marketing controls for formula milks.

Despite differences in financial security, parents spent a similar amount per week on formula milk. At the time of data collection four of the lowest cost milks could be purchased for < £8.50 per tub (Healthy Start allowance at time of data collection), but fewer than 10% of the sample purchased these. Instead, two‐thirds purchased higher priced milks at > £10.50. It appeared that although all parents were struggling with the cost of feeding their baby, this was exacerbated by perceptions or pressures to buy higher cost products that they had been led to believe would be better for their baby, reflecting previous research (Brown et al. 2020; Conway et al. 2023; Vilar‐Compte et al. 2024).

This included parents accessing Healthy/best Start allowance. At the time of data collection, this allowance would cover the cost of the lowest priced milks available. Although it is possible that parents end up using more than one tub per week, may be unable to access these milks (due to location or availability), or may struggle with the additional costs of feeding accessories and preparation meaning that they need to spend more, nearly two‐thirds in this group bought a higher cost brand with just one in six buying the lowest priced milks.

The question arises as to why parents who are struggling to afford milk purchase higher cost products, often pushing themselves into debt, cutting down on their own food or heating or having to adopt practices to make that milk go further (Brown et al. 2026). Advertising and endorsement have a clear influence across the sample, reflecting previous research (Rollins et al. 2023; Conway et al. 2023; Romo‐Palafox et al. 2020). However, it may be having a disproportionate impact on those on lower incomes, with those struggling to afford formula almost twice as likely as those who were not to select milks based on perceived superiority of ingredients or recommendation by health care professionals. This reflects findings in previous research that parents are driven to purchase the perceived best milk for their baby (Brown et al. 2020; Conway et al. 2023; Vilar‐Compte et al. 2024), but this might be exacerbated for parents on a low income. Parents in the survey and interviews were worried about being seen to do the best for their baby, with one mother feeling that buying a “better” milk allowed her to make up for what she could not afford to buy her baby. Even when parents were told by health care professionals that they were all equal in terms of nutritional impact, they didn't believe this or wanted others to see them prioritising spending on formula milk.

Previous research has found that mothers on a lower income can feel increased pressure to show they are a “good mother” (Verduzco‐Baker 2017), worry about doing the ‘best’ for their baby to make up for the impacts of poverty (Rose and McAuley 2019), or feel they face more scrutiny from health and social care professionals than parents from higher socioeconomic backgrounds (Mannay et al. 2018)). Although the analysis did not aim to be comparative, both examples of fear of reprisals or surveillance were from mothers from Black or Asian ethnicities. Systemic racism and differential care have been identified previously in maternity and postnatal care services (Thomson et al. 2022).

Related to the desire to be seen to choose the best product, approximately one in six parents used a brand because it was the one given in hospital on the postnatal/neonatal ward, but this rose to almost one in four of those eligible for Healthy/Best Start. Fewer than one in ten parents who were not struggling gave this as a reason, suggesting brands selected by hospitals are disproportionately affecting parents on a lower income. Some parents felt that postnatal wards would only select the “best” formulas and therefore chose to use these brands, reflecting previous research with parents (Brown et al. 2020; Conway et al. 2023; Vilar‐Compte et al. 2024) and concerns of infant feeding professionals (McNaughton et al. 2025). The formula industry has a long history in attempting to influence health care professionals, often in the guise of educational material to promote specific brands, knowing they will likely in turn influence parents directly or indirectly. The relationship between hospital supply and parent continued choice highlights the need for tighter regulation around this (Baker et al. 2021).

Another reason for continuing to use brands given in hospital, however was the belief that once settled on a specific milk, brand should not be changed, meaning parents continued to purchase higher cost brands. The issue of believing milks should not be changed has been identified previously in research (Conway et al. 2023), but there is no evidence that changing brands causes infant distress. Given recent instances of formula contamination in the UK (Food Standards Agency 2026) and formula shortages in the USA (Tomori and Palmquist 2022) caused significant anxiety in parents, including concerns about needing to change brands, further exploration is needed to build an evidence base around this to help better support and inform parents.

Ethical consumerism also played a role. Some parents described knowing that brands had similar impacts upon infant health and development but chose a brand because of perceived ethical stance, or that it was locally produced in the UK. Consumers are increasingly wanting companies they purchase from to act ethically, including in the infant formula industry (Yang et al. 2021). This has led to criticisms of “green washing” where industry deliberately engages in behaviours seen to be ethical or environmentally friendly in order to increase sales (Kamata et al. 2025), despite significant environmental harms of the breastmilk substitute industry (Pope et al. 2021). The impact of this upon formula brand choice was highlighted in the recent Competition and Markets Authority's (CMA) infant formula and follow‐on formula market report (Competition and Markets Authority 2025). The juxtaposition between a company being perceived as ethical but pricing its products to make a significant profit from parents who are financially struggling is stark.

Availability was also an issue. Concerns arose around supermarket own brands running out or only offered in specific shops. As one mother noted, supplies of lower cost milks reduced with greater publicity that they were nutritionally equivalent. The supply chain must match health promotion messages around choosing infant formula. Transport and accessibility issues have a greater impact on those with a low income (Williamson et al. 2017), highlighting the need to ensure a low‐cost infant formula is readily available across shops. The potential role of a “national formula” milk that would be not‐for‐profit and easily accessible should be explored.

Some mothers who had been unable to breastfeed (or exclusively) described feeling guilty and wanting to make up for this by buying the ‘best’ milk or one perceived to be closest in ingredients or taste. We know that women who stop breastfeeding before they are ready can feel guilt and judgement (Wheeler et al. 2024; Hvatum and Glavin 2017). Marketing capitalises on these emotions, promoting the idea that certain brands are closest to breastmilk (Hastings et al. 2020), driving mothers to purchase them even when they are financially struggling. However, it is notable that mothers who were breastfeeding were actually slightly less likely to purchase a higher cost brand or to purchase a brand for its ingredients compared to those who were exclusively formula feeding. Research may wish to explore reasons for this further, including understanding whether potentially beliefs around breastmilk content or greater exposure to messaging around brand equivalence may be reaching breastfeeding mothers. Given the implications for maternal mental health of not being able to breastfeed and the anxiety and guilt this can carry, how do we ensure that breastfeeding mothers are not feeling pressure from misleading marketing to purchase milks promoted as closer to breastmilk or suited to mixed feeding?

Positively, some parents were aware of equivalence between brands and did not purchase higher cost products. Some parents had received positive messages from health professionals and felt confident to purchase a milk based on it being the lowest cost or conveniently available to them. Research may wish to focus on the experiences of these parents to understand how messaging has reached them and from whom, why they trust it and how they have avoided feelings of pressure to spend more.

Finally, the association of early introduction of solid foods and cow's milk as a drink between financial groups should be noted, especially as many still purchased a higher cost brand. The pattern between early introduction of solids, cow's milk use and deprivation has been found in previous research (Office for Health Improvement and Disparities 2026) but specifically here, in light of association with high levels of purchasing of higher priced formula milk products, it is possible that parents are introducing these potentially harmful behaviours to reduce reliance not only on any formula milk (Brown et al. 2026) but on *higher cost* formula milk products. Further research should explore this, with inclusion of messaging about these potential risks included (Scientific Advisory Committee on Nutrition 2023) in awareness raising around equivalence of infant formula products.

Limitations of this study include the cross‐sectional nature of the study, which cannot be used to determine causality. Further research may wish to build on this study through a longitudinal prospective design. Our sample was self‐selecting and not intended to be representative of the UK general population but instead provides insights into the expenditure and brand decisions of three groups of parents in differing financial situations. There was a significant difference in parent demographic background between the struggling and not struggling groups with those struggling (and particularly those accessing Healthy/Best Start) more likely to be younger, with a lower level of education, have a disability and be neurodiverse. Given the known overlap of these demographic factors with poverty this is unsurprising, but further research may wish to explore intersectionality and experiences of inequality in impact in infant feeding for different diverse population groups.

Many of our sample had experience of breastfeeding and 43% of babies were mixed‐fed, suggesting a slightly higher rate of breastfeeding than the general population (McAndrew et al. 2012). It is possible that mothers who were breastfeeding felt more comfortable taking part in our research, potentially because they may have felt less criticism or stigma around their infant feeding decisions having attempted to breastfeed (Bresnahan et al. 2020). Alternatively, many had not expected to experience breastfeeding difficulties (Ayton et al. 2019) meaning the cost of formula milk came as a shock to their budget increasing both eligibility to participate but also potentially interest in the study.

Online recruitment allowed a diverse sample of parents to be reached, enhancing accessibility. This was supported by sharing study information in face‐to‐face community groups, including those supporting Refugee and Asylum seekers. However, we did not receive any responses from parents from non‐English‐speaking populations. We also recognise the limitations of using a survey with parents on a low income. Research has shown that those on a low income may be more reluctant or face more challenges in taking part (Jang and Vorderstrasse 2019). However, we had a strong response to our study adverts from parents experiencing financial difficulties, perhaps because the study allowed parents an opportunity to share their experiences and have their voices heard. Targeted advertising and working with charity and community organisations enabled us to recruit a sample where over half (54.6%) reported financial difficulties, with one in five claiming Healthy/Best start allowance. The survey format also allowed anonymity which is important for sensitive topics (Braun et al. 2021). Indeed, some parents expressed in the survey that they were happy to complete the survey but did not want to take part in an interview where their identity would become known to the research team.

In conclusion, together the findings show that in line with recommendations of the recent CMA report into infant formula and follow‐on formula (Competition and Markets Authority 2025), stronger regulation (and enforcement) is needed around marketing and promotion of formula milks. There must be wider public understanding that higher priced milks do not offer greater protection for infant health and development. Given that regulation currently does not allow promotion of infant formula milks, this shows that marketing messages of follow‐on, toddler and growing‐up milks are impacting parents' beliefs and purchasing decisions for infant formulas. Fully implementing the International Code of Marketing of Breastmilk Substitutes (World Health Organization 1981) would help to mitigate some of the influence that is affecting parents' spending and disproportionately affecting families experiencing financial difficulties.

## Author Contributions

A.B. was responsible for study conception, study design, data collection, data analysis and draft report writing. C.W. was responsible for study design, data collection, data analysis and draft report writing. V.S., A.G., S.E., S.G., C.G., S.J., H.M., R.P., R.S. and V.T. were responsible for study conception, study design, and draft report writing. All authors read and approved the final manuscript.

## Conflicts of Interest

AB is a volunteer for the Human Milk Foundation and coordinates the Swansea Human Milk Bank hub. SJ is a volunteer for the Human Milk Foundation. VT is a director of First Steps Nutrition Trust. SG is a director of Independent Food Aid Network. SE is a founder of Leicester Mammas. VT is a trustee for First Steps Nutrition Trust and the Medics Lactation community, professional adviser and leader for La Leche League Great Britain, and co‐chairs the Hospital Infant Feeding Network.

## Data Availability

The data that support the findings of this study are available from the corresponding author upon reasonable request.
